# Developmental Origins and Nephron Endowment in Hypertension

**DOI:** 10.3389/fped.2017.00151

**Published:** 2017-06-29

**Authors:** Shari Gurusinghe, Anita Tambay, Christine B. Sethna

**Affiliations:** ^1^Department of Pediatrics, Division of Pediatric Nephrology, Cohen Children’s Medical Center of New York, New York, NY, United States

**Keywords:** hypertension, nephron, fetal origins hypothesis, low birth weight, blood pressure

## Abstract

Primary hypertension continues to be one of the main risk factors for cardiovascular disease worldwide. A stable intrauterine environment is critical for the future development and health of the fetus. The developing kidney has been found to be especially vulnerable during this time period, and epidemiological studies have demonstrated that an adverse *in utero* environment is associated with an increased risk of hypertension and chronic kidney disease. Macro- and micronutrient deficiencies as well as exposure to tobacco, alcohol, and certain medications during gestation have been shown to negatively impact nephrogenesis and reduce one’s nephron number. In 1988, Brenner et al. put forth the controversial hypothesis that a reduced nephron complement is a risk factor for hypertension and chronic kidney disease in adulthood. Since then numerous animal and human studies have confirmed this relationship demonstrating that there is an inverse association between blood pressure and nephron number. As our understanding of the developmental programming of hypertension and other non-communicable diseases improves, more effective preventive health measures can be developed in the future.

## Introduction

Primary hypertension is one of the leading risk factors for morbidity and mortality in the world, and it has been designated as the primary risk factor for the global disease burden (1, 2). Approximately 75 million adults have been diagnosed with hypertension in the United States, and among industrialized countries, it affects 25–35% of individuals globally (3). It is predicted that the number of individuals affected by hypertension will continue to rise and, by 2025, approximately 1.5 billion individuals will be affected (4). Hypertension continues to be the main risk factor for cardiovascular disease (CVD), and current literature states that there is a strong, positive correlation between blood pressure and risk of CVD (5). Although the pathogenesis of hypertension remains unclear, it is evident that the kidneys play a significant role in its development.

Recently, attention has been paid to the contribution of the intrauterine environment to the development of chronic and non-communicable diseases. Epidemiological studies have demonstrated that a poor intrauterine environment is associated with an increased risk of hypertension, chronic kidney disease, and diabetes (6–9). The developing kidney, in particular, has been found to be susceptible to an unstable fetal environment (10). Although multiple factors contribute to the genesis of hypertension, reduced nephron number has been attributed to be a significant contributor (11). In this review, we discuss the various factors that influence nephron endowment. We also highlight key findings on the relationship between nephron number and blood pressure in the pediatric and adult populations.

## Nephron Endowment

Nephrogenesis begins at the gestation age of 9 weeks and is approximately complete by the 36th week (12–14). It is characterized by reciprocal, inductive interactions between the ureteric bud and the metanephric mesenchyme. The metanephric mesenchyme initiates ureteric branching morphogenesis *via* the secretion of growth factors, resulting in the development of the collecting duct system (15–19). Similarly, the branching ureteric bud induces the conversion of mesenchyme to epithelium in the adjacent metanephric mesenchymal cells. These cells are induced to form renal vesicles, which will further differentiate into the comma-shaped bodies and s-shaped bodies ultimately forming a mature nephron. Upon completion of this process, no new nephrons are formed. Therefore, by the end of nephrogenesis, an individual’s entire nephron complement is established (10).

Among humans, the average number of nephrons per kidney is approximately 1,000,000, but there is a significant amount of variation within the human population. Nephron number can range from as low as 200,000 to as high as 2.7 million (20, 21). This variation in nephron number demonstrates the plasticity of the developing kidney, and the significant role one’s environment plays in determining one’s final nephron count.

## Determinants of Nephron Number

**Figure 1:**
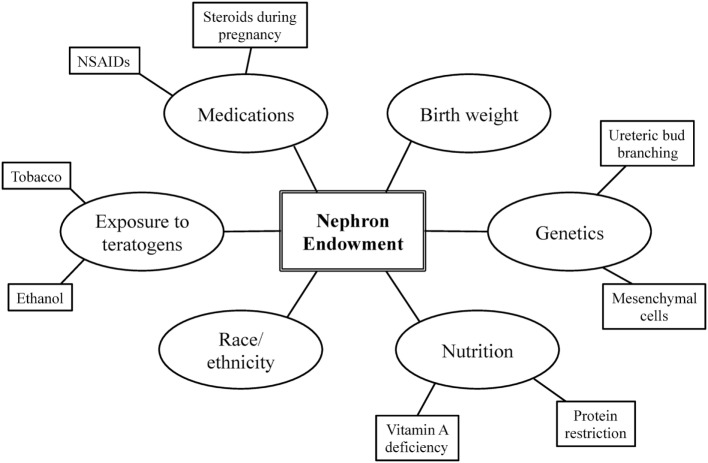
Factors influencing nephron endowment.

Nephron endowment arises from the complex interplay among one’s genetic blueprint, perinatal events, and environmental exposures (Figure 1) (20, 22). A stable intrauterine environment is essential for proper renal organogenesis, and it is becoming more and more evident that the *in utero* environment plays a crucial role in the future development and health of the offspring (10). The following section further describes the relationship among these factors and nephron endowment.

### Birth Weight

Birth weight has been identified as the primary determinant of nephron number (23). Low birth weight due to intrauterine growth restriction or prematurity has been shown to be associated with a reduced nephron complement. When evaluating the coronal sections of kidneys from deceased neonates, Manalich et al. discovered that neonates with lower birth weights (<2,500 g) had significantly fewer glomeruli than those with normal birth weights. In addition, they observed a direct relationship between birth weight and glomerular number and an inverse relationship between glomerular volume and the number of glomeruli. This suggests that individuals with low birth weights are more likely to have fewer, larger glomeruli (24). Similarly, Hughson et al. detected a direct association between birth weight and glomerular number among Caucasian and African-American infants, children, and adults. A 1 kg increase in birth weight was found to be associated with an increase in 257,426 glomeruli. In addition, low birth weight was found to a predictor for fewer glomeruli in their study (23).

In addition, racial and ethnic populations with a high prevalence of low birth weight have been found to have fewer nephrons or smaller kidneys (25–27). Babies born to Australian Aborigines are twice as likely to be of low birth weight compared to non-Aborigines. Hoy et al. discovered that Australian Aboriginal subjects had approximately 404,000 fewer glomeruli than non-Aboriginals and a significantly larger mean glomerular volume (25). They also observed a strong association between adult height and glomerular number. These findings further corroborate the relationship between birth weight and nephron number within this population, as birth weight is a strong predictor of adult height.

### Genetic Factors

Numerous mouse models have demonstrated that inadequate ureteric bud branching results in decreased nephron number. Null mutations in genes implicated include those regulating the formation and function of: epidermal growth factors, fibroblast growth factors (e.g., *fgf7* and *fgf10*), glial cell-derived neurotrophic factor (*gdnf, c-ret*, and *gfrα*), hepatocyte growth factor (e.g., *hgf* and *c-met*), transforming growth factor β (e.g., *tgfβ2* and *tgfβ3*), and signal transduction proteins from the Wnt family (28–32). For purposes of this review, we have chosen to highlight two genes whose physiological implications have been demonstrated through studies on human subjects.

The Six2 gene has emerged as a key player in kidney development. It is expressed throughout renal organogenesis in undifferentiated mesenchymal cells and encodes a homeodomain transcriptional regulator (33). Its continual expression is needed to maintain the nephron progenitor population (34, 35). Kobayashi et al. demonstrated that Six2+ expressing cap mesenchymal cells are multipotent nephron progenitors that give rise to the multiple domains of the nephron (34). Its inactivation results in the reduction of the number of progenitor cells, and these cells autonomously maintain its own cell population (34, 35). The knock out of the Six2 gene in mice models was found to result in the formation of ectopic renal vesicles (35). Furthermore, Weber et al. discovered mutations within the Six2 gene among a subset of patients with renal hypodysplasia (36). Therefore, mutations, which affect the Six2 gene product, can adversely affect nephrogenesis.

Interestingly, genetic variants that increase nephron number have also been identified. El Kares et al. identified a variant of the ALDH1A gene, rs7169289(G), that is associated with an increased total kidney volume in newborns when adjusting for body surface area. The ALDH1A gene is involved in the metabolism of retinoic acid, and infants who were homozygous for this variant were found to have higher umbilical cord blood levels of retinoic acid (37).

### Nutrition

Maternal nutrition plays an essential role in the development of the fetus. The developing kidney is especially vulnerable to the effects of a poor maternal diet, and maternal malnutrition during pregnancy has often been proven to be associated with suboptimal renal phenotypes among her offspring (10). Macro- and micronutrient deficiencies as well as restricted caloric intake have been demonstrated to impair kidney development during gestation (10, 22).

Protein restriction during gestation significantly reduces nephron number (38–41). Woods et al. discovered that male protein-restricted rats had 25% less nephrons than protein replete controls as well as a higher mean arterial blood pressure in adulthood. Renal hyperfiltration was also observed among subjects, as protein-restricted offspring had a higher individual nephron glomerular filtration rate. Interestingly, the subjects’ intrarenal renin mRNA, renin concentration, and immunostaining for renin were reduced during nephrogenesis. Subsequently, their intrarenal angiotensinogen II levels were also reduced throughout nephron development. These results imply that RAS has an additional role in the regulation of blood pressure. In addition to maintaining blood pressure in adulthood, it influences the final nephron number during nephrogenesis. Therefore, the RAS system may act as a mediator in the relationship between protein restriction and nephron number (40). A high protein diet, however, was not found to influence nephron endowment (42).

Micronutrients also significantly impact renal organogenesis (10, 22). Gilbert proposed that vitamin A is largely responsible for significant variations in nephron number among humans. Vitamin A serves as a ligand to c-RET, c-ret tyrosine kinase receptor (43). This receptor plays a critical role in early kidney development as it is involved in the initiation of ureteric branching. Maternal iron and zinc deficiencies have also been found to reduce nephron number and increase systolic blood pressure in adult offspring. However, the mechanism behind these associations is less understood (10). Overall, there are a variety of nutrient deficiencies that result in the same structural renal phenotype. Therefore, a reduced nephron number may serve as an adaptive response from the developing kidney during times of environmental stress (10).

### Teratogens

The detrimental effects of chronic alcohol exposure to the fetus during pregnancy have been well documented. However, the effects of acute prenatal alcohol exposure are less understood, especially its influence on the developing kidney. Gray et al. investigated the effects of acute prenatal ethanol exposure on nephron endowment in rats. Either ethanol or saline was administered to Sprague-Dawley rats on embryonic days 13.5 (E13.5) and 14.5 (E14.5). Ethanol exposed rodents were found to have a 10–20% reduction in nephron number as well as a higher mean arterial blood pressure. In addition, E15.5 rats were found to have a reduced gene expression of significant regulators of ureteric branching morphogenesis (GDNF, FGF7, Wnt11, TGFβ2, and TGFβ3). Fewer ureteric branch points and tips were also found in embryonic kidneys that were cultured in media containing ethanol (44).

Tobacco exposure is another established teratogen, which has been demonstrated to be negatively associated with birth weight and positively associated with blood pressure (45, 46). Taal et al. conducted a population-based prospective cohort study of 1,072 mothers and children to investigate the association between prenatal cigarette exposure and kidney volume. Mothers who only smoked during the first trimester and those who smoked throughout their pregnancy were followed. A consistent relationship between smoke exposure and kidney volume was not observed among the offspring of mothers who smoked solely during the first trimester. However, a dose-dependent relationship between kidney volume and tobacco exposure was observed among the offspring whose mothers smoked throughout their pregnancy. Those who smoked more than 10 cigarettes a day had a smaller total fetal kidney volume than those who smoked less than five cigarettes a day (47). These results once again imply the importance of a stable intrauterine environment as disturbances may predispose an individual to cardiovascular and renal diseases.

### Medication

Elevated levels of maternal corticosteroids during pregnancy have been shown to be associated with a low nephron complement and higher blood pressure among her offspring (22). Interestingly, both the duration and timing of the exposure determine the severity of the phenotype and only 2 days of exposure is needed to produce a nephron deficit. Administration of dexamethasone (DEX), a synthetic glucocorticoid, during embryonic day E15/16 or E17/E18 was found to decrease glomerular number and increase blood pressure in rat models. Similarly, DEX exposure during day E19/E20 or E26-28 was also found to result in hypertensive sheep offspring with a nephron deficit. However, administration of DEX before or after these time periods was not found to affect glomerular number or blood pressure. The time periods when animals were most susceptible to DEX were during times of kidney development, specifically during ureteric branching morphogenesis. Singh et al. proposed that DEX reduces nephron number by inhibiting/slowing ureteric branching. They reported that DEX exposure decreased the expression of GDNF (a promoter of ureteric branching) and increased the expression of inhibitors, BMP-4 and TGF-β1 both *in vitro* and *in vivo* (48). Ureteric branching morphogenesis is believed to be a significant process in establishing the final nephron complement as each ureteric tip induces the formation of new nephrons. Therefore, by inhibiting ureteric branching, DEX indirectly reduces nephron number (48).

Exposure to non-steroidal anti-inflammatory drugs has been shown to have variable effects on one’s nephron number (22). Rodents that were exposed to cyclooxygenase-2 inhibitors from gestation to 3 weeks postbirth were found to have a significantly reduced glomerular size. Interestingly, if exposure occurred solely during gestation, no effect on glomerular size was observed (49).

## Developmental Origins of Health and Disease

One of the initial proponents of the fetal origins of disease was Dr. David Barker. In the 1980s, Barker and colleagues discovered an inverse relationship between birth weight and coronary artery disease mortality rates in a cohort of men and women born in Hertfordshire, United Kingdom. They observed a twofold increase in mortality rates when comparing individuals with the lowest birth weight with those with the highest birth weight (50, 51). Barker eventually proposed that fetal undernutrition during middle to late gestation “programs” coronary artery disease in adulthood (52). Future epidemiological studies supported the Barker hypothesis, demonstrating the importance of one’s intrauterine environment in determining one’s future health (6–9). These findings further shed light on the non-hereditary component of chronic diseases.

The theory of “developmental programming” or the “developmental origins of health and disease,” states that environmental influences during sensitive periods of development can result in permanent alterations of function and structure of an organism (53). In order to increase its chances of surviving, the fetus may undergo structural and functional changes during gestation at the expense of its future health. Therefore, an adverse intrauterine environment can predispose an individual to an increased risk of hypertension and cardiovascular or renal disease (6–9). The developing kidney has been shown to be especially vulnerable to a suboptimal *in utero* environment (10). As described previously, in addition to one genetic disposition, macro- and micronutrient deficiencies and exposure to teratogens and certain medications during gestation have been found to reduce one’s nephron endowment (10, 22, 44, 48). Interestingly, each of these different environmental signals elicits a similar renal phenotype in the offspring.

**Figure 2:**
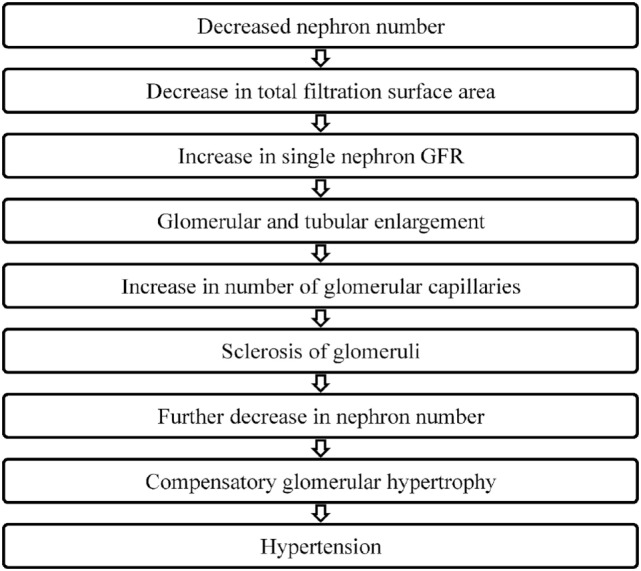
How does nephron number lead to hypertension?

In 1988, Brenner et al. put forth the hypothesis that a low nephron number is a risk factor for adult hypertension (Figure 2). They proposed that a reduction in the total filtration surface area of the kidneys is associated with a compensatory increase in the single nephron glomerular filtration rate. In response to a reduced nephron complement, adaptive structural changes occur within the nephron including glomerular and tubular enlargement and an increase in the number of glomerular capillaries. Consequently, the afferent arteriole dilates while the efferent arteriole constricts resulting in an increase in the glomerular capillary pressure. This reduction in the afferent arteriolar resistance allows for an increased transmittance of systemic blood pressure into the glomerulus. Simultaneously, other physiological changes occur that also contribute to the development of hypertension including increased salt reabsorption, higher volume strokes and cardiac output and resetting of the pressure-natriuresis curve. Pathological changes such as podocyte detachment and tuft adhesion to Bowman’s capsule have been noted in sclerosed kidneys compensating for hyperfiltration (e.g., secondary to vesicoureteral reflux) (54). Over time, this sclerosis of the glomeruli fuels a vicious cycle resulting in a decreased nephron number, the compensatory glomerular hypertrophy, and the progressive hypertension and chronic kidney disease (55–57).

## Nephron Number and Hypertension

Since initially hypothesized over 30 years ago, there have been a number of animal and human studies that have sought to determine the relationship between nephron endowment and hypertension.

### Animal Studies

Through animal studies, it is becoming increasingly clear that a relationship exists between nephron number and hypertension. A mouse model of prematurity found that those delivered 1 and 2 days early had 17.4 and 23.6% fewer nephrons, respectively, compared to full-term mice, and the premature mice subsequently developed hypertension (58). In addition, a number of genetic knock out mice models associated with lower nephron number have demonstrated an inverse association with blood pressure (59–61).

### Low Birth Weight and Nephron Number in Humans

Human epidemiological studies have used birth weight as an indicator of fetal nutrition status and therefore a surrogate for lower nephron endowment. Systematic reviews and meta-analyses of the literature have consistently shown an inverse relationship between birth weight and blood pressure. Law et al. reviewed 34 cohort studies published before 1996. After adjustment for body mass index, regression coefficients of birth weight indicated a negative association with systolic blood pressure in 26 studies, indicating a 2–3 mmHg/kg reduction in systolic blood pressure in children and 2–4 mmHg/kg reduction in adults with increasing birth weight (62). In a review by Huxley et al. of 45 pediatric and adult studies published between 1996 and 2000, the majority (33 studies) reported a negative association between birth weight and systolic blood pressure. In aggregate, an 1 kg increase in birth weight was associated with an 1–2 mmHg/kg decrease in systolic blood pressure (63). A review by Adair et al. of 2000–2005 publications of adult studies remains consistent with the earlier ones. Of the 28 cohort studies, 25 studies found an inverse association; however, not all were adjusted for BMI (64). More recently, a meta-analysis of 27 studies conducted between 1995 and 2012 found that low birth weight (<2,500 g) compared with birth weight greater than 2,500 g was associated with an increased risk of hypertension (odds ratio 1.21; 95% confidence interval 1.13, 1.30) (65).

The relationship between birth weight and blood pressure in childhood is more complicated, and studies published since 2000 have shown inconsistent results muddied by poorly powered studies focusing on specific populations. There is also some evidence that the inverse relationship between blood pressure and birth weight becomes more pronounced as the age of the study population increases, although this finding is weak and may not be statistically significant (66). Studies of adolescents show conflicting results, and in neonates, some studies reported a positive relationship between blood pressure and birth weight (62). Rahiala et al. showed that birth weight was not an independent determinant of blood pressure in 100 children in Finland using ambulatory blood pressure monitoring (67). However, four other studies with larger sample sizes supported the inverse relationship of birth weight and ambulatory blood pressure in children (68–71). Though low birth weight does appear to be associated with hypertension, the physiological and molecular mechanisms establishing causality remain to be elucidated.

### Impact of Ethnic and Racial Factors

There is a significantly higher prevalence of hypertension in African-Americans, which is likely multifactorial in etiology. African-Americans are also at higher risk for being low birth weight. This suggests that being low birth weight could be one of the factors implicated in predisposing African-Americans to higher rates in hypertension later in life (23).

Since most of the studies in this area focused on Caucasians, there is little published on the African-American population. Hulman et al. evaluated 137 urban African-American adults and found no relationship between birth weight and adult blood pressure (72). Donker et al. looked at a biracial sample of 1,446 children aged 7–11 years and found evidence that low birth weight is a determinate of high blood pressure only in African-American males. However, the association was lost when multivariate analyses were done (73). A study by Rostand et al. analyzed 262 Caucasian and African-American 5-year-old children. An inverse relation was found for Caucasian children, but, surprisingly, a positive relationship was found for African-American children (74). However, an inverse relationship between birth weight and blood pressure was found in the longitudinal Bogolusa Heart Study and there were no racial differences found (75).

Interestingly, hypertension is also highly prevalent among Australian Aboriginals and it appears that a reduced nephron complement mediates the relationship between low birth weight and hypertension within this population (25). Hoy et al. reported that Aboriginals were endowed with fewer and larger glomeruli than non-Aborigines. In addition, as expected, Aborigines with a history of hypertension had significantly fewer glomeruli than those who did not. When evaluating the relationship between kidney volume and blood pressure within this population, the investigators reported that renal volume was inversely associated with blood pressure in both Aboriginal children and adults.

### Postnatal Influences on Nephron Number

Low birth weight has been shown to impact the nephron number at birth; however, numerous factors affect nephron number throughout one’s lifetime. Environmental factors such as weight gain and aging later change nephron number and therefore risk (76, 77). However, studies evaluating these factors have been difficult to conduct in humans, as there are no accurate methods accounting for nephron number that leave the kidney undamaged.

Nephrons are hypertrophied atrophy sooner, therefore decreasing the nephron number even more so (78). Though it is commonly said that reduction in glomerular number occurs naturally with age, far greater reduction has been found to occur in hypertensive patients (79). Keller et al. found that the mean glomerular number for hypertensive patients was half that of the normotensive patients (11), though the mean glomerular volume was 133% greater. In this same study, kidney samples were also analyzed for histological evidence of hypertension. Even when including glomeruli obliterated due to sclerosis, hypertensive patients had significantly lower glomerular number than normotensive patients.

## Conclusion

There is an increasing body of literature demonstrating the relationship between nephron number and hypertension. These studies show that there is an inverse relationship between nephron endowment and hypertension. As this relationship is established, factors that contribute to determining nephron number are being elucidated. Genetics clearly plays a role in nephron endowment, one that possibly explains racial differences in nephron number at birth. Environmental factors such as birth weight, body size, and age also have an impact. Having a way to assess for life-long risk of renal and CVD could be instrumental in initiating preventive health measures earlier in life. Like genetics, low birth weight is a predetermined risk factor that could be utilized for risk stratification and thus early initiation of preventive health measures. Recent advances in accurate and precise estimates of glomerular number will improve our understanding of the factors contributing to nephron endowment and its implications for future health.

## Author Contributions

SG, AT, and CS contributed equally to the writing of this manuscript.

## Conflict of Interest Statement

The authors declare that the research was conducted in the absence of any commercial or financial relationships that could be construed as a potential conflict of interest.
